# Immobilization of *Tth*LPMO9G on Carbon Felt for Potential Electrochemical Applications

**DOI:** 10.1021/acsomega.5c02275

**Published:** 2025-05-12

**Authors:** Koar Chorozian, Anthi Karnaouri, Theodora Kouvarati, Antonis Karantonis, Evangelos Topakas

**Affiliations:** † IndBioCat Group, Biotechnology Laboratory, School of Chemical Engineering, 68994 National Technical University of Athens, Athens 157 72, Greece; ‡ Laboratory of General and Agricultural Microbiology, Department of Crop Science, 68995 Agricultural University of Athens, Athens 11855, Greece; § Laboratory of Physical Chemistry, School of Chemical Engineering, 68994 National Technical University of Athens, Athens 157 72, Greece

## Abstract

This study explores the entrapment, immobilization, and direct electron transfer-type bioelectrocatalysis mediated by *Tth*LPMO9G on carbon-based electrode materials, focusing on carbon felt (CF) due to its high conductivity, chemical stability, and large surface area. At first, entrapment of an LPMO from Thermohelomyces thermophila,*Tth*LPMO9G was achieved using Nafion-coated carbon fibers. At the next step, CF electrodes were chemically oxidized to introduce carboxyl groups, quantified by conductometric titration, and used for covalent immobilization of the enzyme. The immobilization process for *Tth*LPMO9G was optimized, and the catalytic activity was assessed based on cellulose oxidation. The success of the immobilization process was evaluated using three parameters: yield (%), efficiency (%), and %recovery (%). Electrochemical studies, including cyclic voltammetry (CV) and Fourier-transform alternating current voltammetry (FTacV), were performed to evaluate *Tth*LPMO9G’s electrochemical activity. Immobilized LPMO activity was detectable only through the more sensitive FTacV. Direct electron transfer (DET) from the electrode to the enzyme active site remains a challenge. This work provides insight into the limitations of the studied strategies in LPMO-based electrocatalysis, thus offering guidance for improving immobilization process and electrochemical integration in future applications targeting DET bioelectrocatalysis.

## Graphical abstract

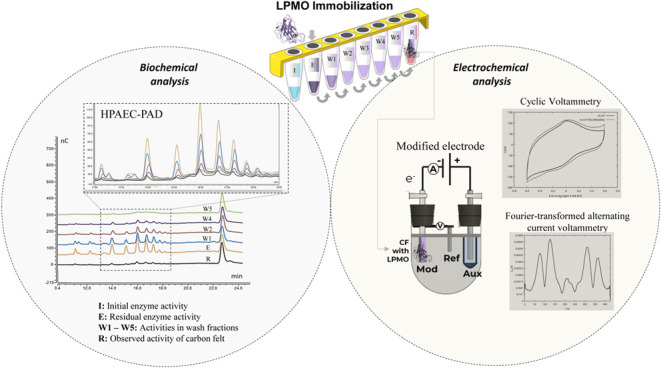

## Introduction

The search for sustainable and efficient ways to valorize biomass has highlighted enzyme immobilization as a practical method to improve the industrial use of biocatalysts. Among these, lytic polysaccharide monooxygenases (LPMOs) have gathered significant attention due to their unique mechanism of oxidative cleavage of recalcitrant polysaccharides, such as cellulose. First described by Vaaje-Kolstad et al.,^1^ LPMOs revolutionized our understanding of polysaccharide degradation by introducing an oxidative mechanism that destabilizes glycosidic bonds. This mechanism, facilitated by a copper ion active site coordinated by the characteristic histidine brace, and promoted by electrons provided by a reductant, enables LPMOs to overcome the energy barrier for glycosidic bond cleavage in insoluble polysaccharides.^2,3^ LPMO catalysis follows two suggested pathways: (a) O_2_-driven R-H + O_2_ + 2e^–^ + 2H^+^ → R–OH + H_2_O, where Cu­(II) is reduced by two electrons to activate O_2_ for hydroxylation,^1^ and (b) H_2_O_2_-driven R–H + H_2_O_2_ → R–OH + H_2_O, where Cu­(I) reacts directly with H_2_O_2_, requiring only an initial reduction to sustain multiple catalytic cycles.^4^ Early studies identified the chitin-binding protein CBP21 from Serratia marcescens as an LPMO capable of cleaving β-(1 → 4) glycosidic bonds in chitin through oxidation. LPMOs have since been identified across diverse organisms, including bacteria, fungi, viruses, and invertebrates, and exhibit activity on a broad range of carbohydrate substrates.^5,6^

Oxidoreductases, such as glucose oxidase, have been successfully immobilized on conductive materials, including carbon felt (CF)^7^ and graphene oxide,^8^ with the latter enabling effective electron transfer to the enzyme active site. Several electrode-based approaches for studying LPMOs in electrochemical systems have been explored in the literature, thus highlighting the potential for integrating LPMOs into such systems for efficient electron provision.^9–12^ Immobilization of LPMOs presents an attractive avenue for their application in biorefineries and beyond, offering improved enzyme stability, reusability, and operational control. Electrochemistry is employed both to investigate the activity and mechanism of LPMOs’ action and to harness its advantage of providing electrons directly to the enzyme without the need for an external reductant, enabling bioelectrocatalysis. Key parameters such as enzyme, reducing agent, and copper loading concentration, as well as reaction time and cellulose substrate levels, have been fine-tuned to prevent LPMO inactivation in solution-based systems.^13,14^ However, these optimized conditions need to be further tailored for immobilization, accompanied by the appropriate control reactions to ensure accurate interpretation of immobilized LPMO activity. Additionally, immobilizing these enzymes poses challenges due to their reliance on electron donors to sustain catalytic activity. Moreover, given the surface-exposed nature of the enzyme’s active site, a defined spatial orientation is required during immobilization to maintain sufficient substrate accessibility. Research on LPMO immobilization is limited, with Cai et al.^15^ being the only reported study to date, where the synergism of lytic polysaccharide monooxygenases with lichenase was explored. The limited understanding of electron transfer mechanisms in natural systems,^16^ coupled with the lack of exploration in LPMO immobilization, hampers broader applications, particularly in electrode-based systems for electron provision. In addition, the use of insoluble substrates further limits enzyme–substrate interactions, requiring strategies to improve accessibility.

Carbon-based textile materials have low surface energy and are chemically stable, corrosion-resistant, and conductive, making them suitable and cost-effective for use in bioelectrochemical applications.^17–19^ One of the most important applications for carbon-based textiles is the production of electrodes. However, their hydrophobic properties limit their use in aqueous systems and composites due to poor wettability and weak adhesion.^20^ To overcome these challenges, several methods including oxidation have been used to introduce reactive groups like carboxylates (−COOH).^21,22^ Such modifications enable the covalent attachment of biomolecules, including enzymes. In this context, functionalized CF could serve as a promising material for exploring the immobilization of LPMOs, with the potential to enhance their stability and activity in bioelectrochemical setups.

In this study, we initially standardized the investigation of the electrochemical activity of an LPMO from Thermohelomyces thermophila, namely *Tth*LPMO9G,^23,24^ using entrapment methods such as Nafion coating, a polyelectrolyte known for its ability to trap enzymes and facilitate electron transfer.^25^ *Tth*LPMO9G, known for its thermal stability and C1-regioselectivity, cleaves glycosidic bonds by oxidizing the C1 carbon atom of the glucose unit, resulting in the formation of a carboxyl group at the nonreducing end of the cleaved polysaccharide.^23^ Nafion was selected as the initial entrapment substrate to provide a literature-based reference point, as it is the only method previously reported for electron transfer studies involving AA9 LPMOs in electrochemical setups.^9^ Building on these findings, we focused at the following step on *Tth*LPMO9G immobilization on modified CF, which is able to facilitate electron transfer to the enzyme’s copper-active site. This study addresses key challenges in LPMO immobilization, including enzyme entrapment, optimization of immobilization conditions, and interactions with carbon-based conductive materials.

## Results and Discussion

### Evaluating *Tth*LPMO9G Kinetics under Immobilization-Related Conditions

LPMOs have great potential in biorefineries due to their ability to degrade cellulose efficiently. Covalently immobilizing or entrapping LPMOs can enhance their stability and reusability but presents challenges due to their reliance on electron provision for active site reduction, the surface-exposed nature of their active site, the potential instability of their copper coordination^26^ and sensitivity to auto-oxidation.^4,27^ To optimize immobilization or entrapment conditions for *Tth*LPMO9G, we first assessed the free enzyme’s activity under varying enzyme and electron donor concentrations using phosphoric-acid swollen cellulose (PASC) as the substrate. These conditions are chosen to replicate those that will be applied later to the enzyme immobilized on conductive materials functioning as electrode systems, to allow direct comparisons between the free and immobilized enzyme. This evaluation focused on two critical aspects: (i) ensuring sufficient enzyme loading for immobilization is of pivotal importance and (ii) understanding enzyme performance under conditions where electrons are supplied by the electrode is particularly challenging, as this setup provides a more intense and potentially harsher electron supply compared to low-concentration small-molecule reducing agents. As shown in Figure 1A, the production of oxidized cello-oligosaccharides increased only at lower enzyme concentrations but plateaued at higher concentrations when 5 mM ascorbic acid was used as the reducing agent after 1 h—incubation in the presence of 0.2% PASC.^13^ For immobilization, where high enzyme loading is desirable, these findings emphasize the importance of fine-tuning conditions to avoid activity loss, and proper dilutions when estimating immobilization yield. It is essential to accurately characterize the free enzyme present in the remaining solution fractions, as it represents the unbound portion of LPMO, often present at high concentrations that can lead to autoxidation and reduced catalytic efficiency. The influence of electron donor concentration was also explored (Figure 1B,C), with ascorbic acid serving as the reducing agent. For LPMOs, the optimal electron supply corresponds to an ascorbic acid concentration range of 0.1–0.5 mM; however, achieving precise control with electrodes at such low levels is challenging. To address this, higher ascorbic acid concentrations (0.2–5 mM) were tested to replicate conditions of intense electron supply that might occur in a conductive system with an electrode (Figure 1B,C). Using increased ascorbic acid concentrations, we evaluated the enzyme’s activity on PASC by quantifying C1-oxidized products. A control reaction with 4 mM ascorbic acid and PASC, in the absence of enzyme, showed no detectable C1-oxidized products (Figure S1). However, it is important to note that enzyme inactivation in this setup could occur due to H_2_O_2_ generated by the oxidation of ascorbic acid.^14,24^

**Figure 1:**
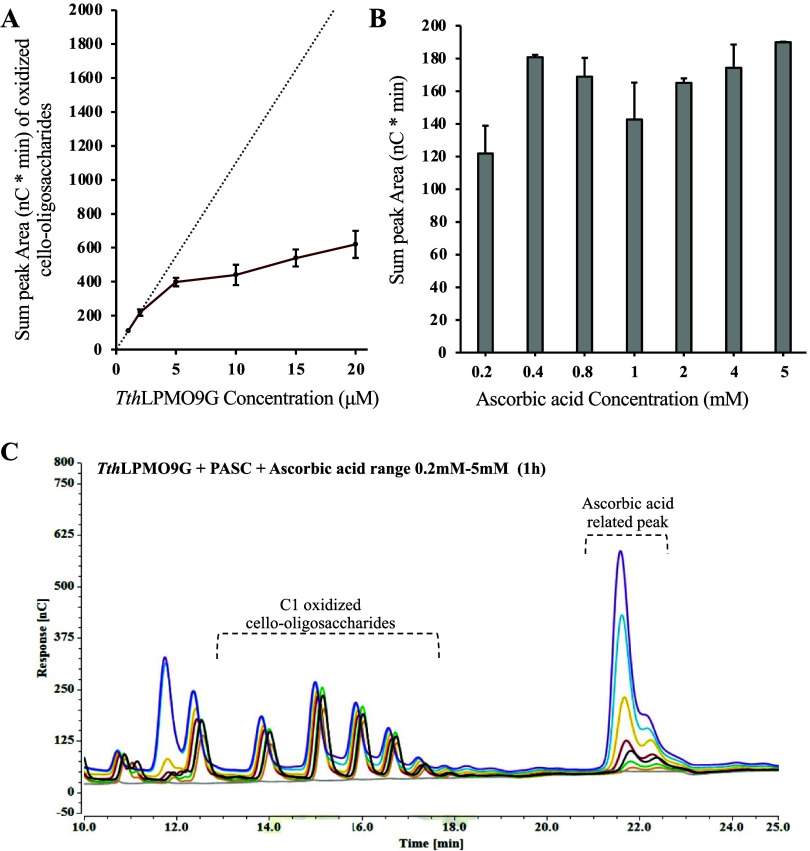
Evaluation of *Tth*LPMO9G (free enzyme) activity under varying enzyme concentrations and electron donor levels, using PASC (0.2% w/v) as the substrate after 1 h incubation. (A) Quantification of oxidized cello-oligosaccharides produced by *Tth*LPMO9G at varying enzyme concentrations in the presence of 2 mM ascorbic acid, measured using HPAEC-PAD. The dotted line represents the theoretical linear correlation between enzyme concentration and product formation, while the solid red line represents the observed data. At higher enzyme concentrations, the production of oxidized cello-oligosaccharides deviates from linearity possibly due to enzyme autoxidation and inactivation. (B) Total C1-oxidized cello-oligosaccharide production quantified as the sum of peak areas from HPAEC-PAD analysis at varying concentrations of ascorbic acid (0.2–5 mM), of 20 μM enzyme concentration. Line colors correspond to increasing concentrations of ascorbic acid: 0 mM (gray), 0.2 mM (orange), 0.4 mM (green), 0.8 mM (black), 1 mM (magenta), 2 mM (yellow), 4 mM (turquoise), and 5 mM (purple). Increasing electron donor concentrations enhanced product formation, with a plateau observed at higher concentrations. (C) Chromatograms obtained from HPAEC-PAD show the production of C1-oxidized cello-oligosaccharides. Peaks corresponding to oxidized products are visible in the range of 13–19 min, with an ascorbic acid-related peak observed at 22 min. This figure highlights the dependence of *Tth*LPMO9G activity on enzyme concentration and electron donor levels, providing insights for optimizing enzyme performance under conditions mimicking potential electrochemical applications. The control reaction, represented by the gray chromatogram, contains no ascorbic acid, with a 20 μM enzyme concentration.

### Electrochemical Performance of Entrapped *Tth*LPMO9G on Nafion-Coated Carbon-Based Materials

In a previous study, LPMOs were successfully entrapped in a Nafion matrix on glassy carbon electrodes, demonstrating evidence of direct electron transfer between the enzyme and the electrode.^9^ Notably, no substrate was present in this system, as the setup was designed solely to observe the electrochemical activity of the enzyme, without involving the oxidation of cellulose. Building on this, the current study evaluated the entrapment and electrochemical activity of *Tth*LPMO9G on various carbon-based electrode substrates, including glassy carbon, carbon fiber bundles and CF, where electrochemical activity refers to the enzyme’s ability to accept and donate electrons. To increase the surface area, the carbon-based electrode substrates were coated with a commercial highly conductive colloidal graphite-based spray before enzyme entrapment. To increase the surface area, the carbon-based electrode substrates were coated with a commercial, highly conductive colloidal graphite spray prior to enzyme entrapment. *Tth*LPMO9G was then deposited onto the substrates, dried, and embedded in a Nafion layer. The enzyme’s electrochemical activity was assessed by cyclic voltammetry (CV), which revealed redox peaks attributed to *Tth*LPMO9G. Control experiments confirmed the system’s reliability: Nafion blanks (F-Nafion) showed no redox peaks (Figure 2), while positive controls containing potassium ferricyanide (F-Nafion-K_3_[Fe­(CN)_6_]) and ferrocene (F-Nafion-Ferro) exhibited clear, reversible redox peaks. These results demonstrate that the Nafion matrix supports electron transfer from redox-active species to the electrode, validating its use for further enzymatic studies. The enhanced surface area provided by the conductive graphite coating likely contributed to efficient enzyme entrapment and electron transfer. These observations confirm that the observed peaks were specifically attributed to the enzymatic activity. Among the tested configurations, the carbon fiber bundles coated with conductive graphite and Nafion proved to be the most effective for achieving electrochemical activity, demonstrating their potential for bioelectrochemical applications, compared to the glassy carbon and CF (data are not shown).

**Figure 2:**
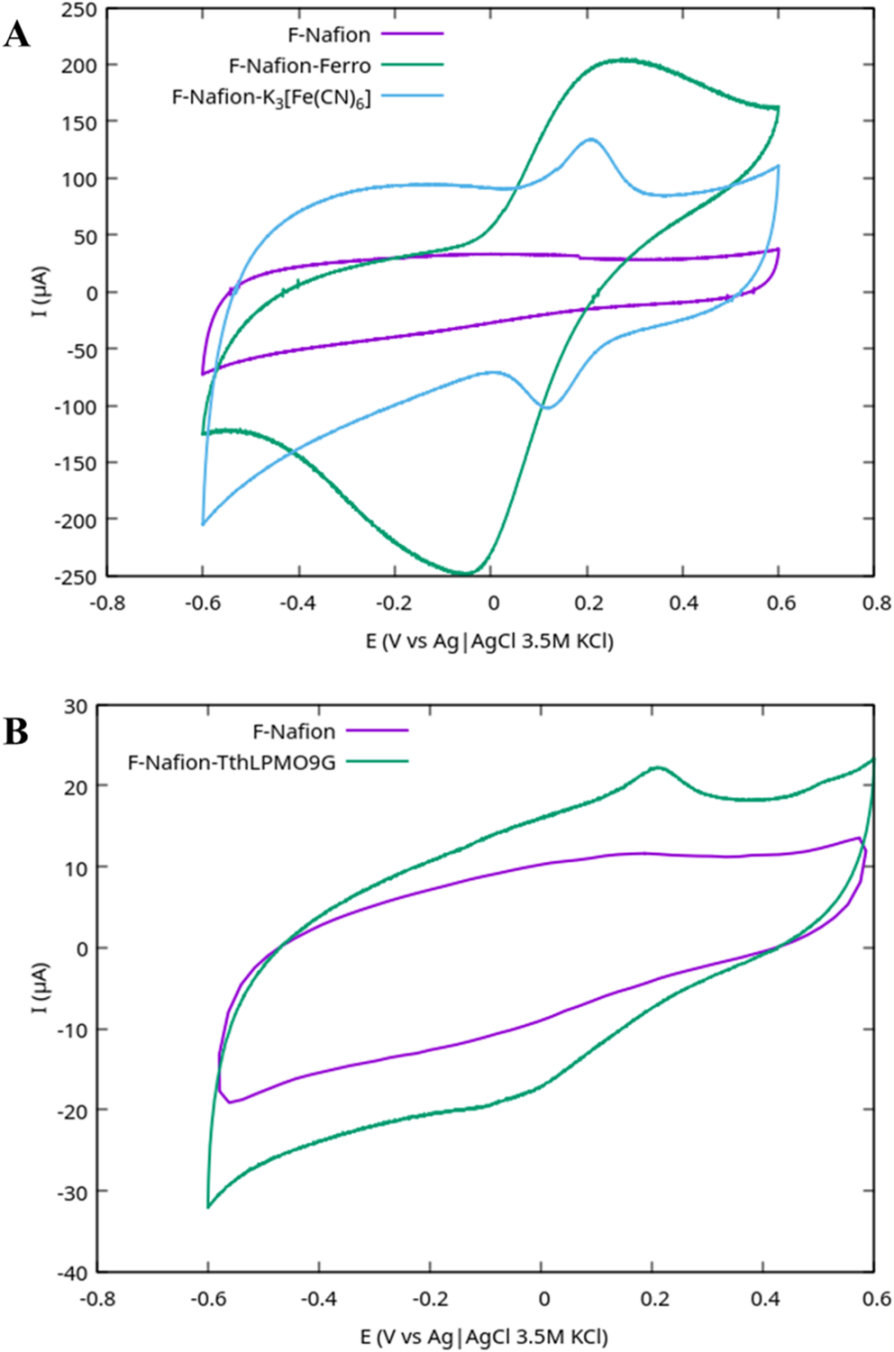
Cyclic voltammograms of carbon fiber bundles coated with DUE-CI ELECTRONIC Graphite Spray N-77 and Nafion film, recorded in the potential range of [−0.6 to 0.6 V]. (A) Nafion with ferrocene and Nafion with potassium ferricyanide were both tested in 0.5 M Na_2_SO_4_ (pH adjusted to 7–8 with ammonia), scan rate 50 mV/s. (B) Nafion with immobilized *Tth*LPMO9G was tested in sodium acetate buffer (pH 4–5), scan rate 10 mV/s. Blank Nafion is shown as the control.

### Modification of Carbon Felt

As described above, carbon fiber bundles coated with conductive graphite and embedded in a Nafion matrix showed evidence of direct electron transfer (DET) for *Tth*LPMO9G. The Nafion matrix provided an effective initial platform to demonstrate the enzyme’s ability to exchange electrons in an electrochemical system. However, the goal of this study was to develop a system where the enzyme could not only exchange electrons but also perform its catalytic function, such as the oxidation of cellulose. While the Nafion-based setup worked well for assessing *Tth*LPMO9G electrochemical activity, its design inherently restricted substrate access to the enzyme’s active site, which would be necessary for catalytic activity involving insoluble substrates like cellulose. To address this limitation and enable direct electron transfer while maintaining active site accessibility, the study transitioned from enzyme entrapment to covalent immobilization. This approach was intended to overcome the constraints of the entrapment system and meet the specific demands of bioelectrochemical applications.

CF is widely used in bioelectrochemical applications for enzyme immobilization. Immobilization generally involves covalent or noncovalent attachment of enzymes to the electrode surface, ensuring they remain fixed and able to facilitate efficient electron transfer.^28^ The CF used in this study (AvCarb G200, PAN-based) displayed good conductivity and a fibrous structure that made it suitable for bioelectrochemical systems. Scanning electron microscopy (SEM) revealed its dense network of fibers with an average diameter of 8.74 μm (Figure S2). These characteristics, along with its chemical and electrochemical stability in aqueous environments, made it a strong candidate for *Tth*LPMO9G immobilization. The aim was to design a system where the enzyme could form covalent bonds with the conductive material, ensuring stable attachment while maintaining access to the active site. CF was chosen as the optimal substrate because of its high surface area, fibrous structure, and ability to provide a stable platform for long-term applications (Table S1).

The chemically inert and hydrophobic nature of CF necessitated surface modification to enable effective enzyme immobilization.^7^ Chemical oxidation using a sulfuric and nitric acid mixture successfully introduced carboxyl (−COOH) groups onto the CF surface. Conductometric titration confirmed the presence of reactive groups, with optimized oxidation conditions (64 h at 80 °C) yielding up to 2.1 mol −COOH/g CF as indicated in Table 1. Some samples of CF were tested at increasing oxidation times to evaluate the impact of time on the mol −COOH/g CF yield. Higher reaction temperatures (120 °C) did not significantly enhance carboxyl group content, suggesting that oxidation duration plays a more critical role than temperature in surface functionalization (data not shown).^28^ The carboxylated CF was subsequently treated with 1-ethyl-3-(3-(dimethylamino)­propyl) carbodiimide (EDC) and *N*-hydroxysuccinimide (NHS) in MES (2-(*N*-morpholino)­ethanesulfonic acid) buffer (0.2 M, pH 5.0). This reaction converted the carboxyl groups into reactive intermediates, enabling covalent bonding with primary amine groups on the enzyme as previously described.^29^ Initially, the protocol for covalently immobilizing enzymes was standardized using GH11 xylanase, as its activity can be evaluated with a simpler and more straightforward assay. This served as a basis for adapting the protocol to *Tth*LPMO9G, which requires more complex evaluation.

**Table 1:** Carboxyl Group Density of Oxidized CF Samples

sample	oxidation time (h)	carboxyl content (mmol −COOH/g CF)
CF_a_	8	0.206
CF_b_	16	0.532
CF_c_	18	0.518
CF_d_	64	1.806
CF_e_	64	2.107

### Standardization of Immobilization Using GH11 Xylanase

To standardize the immobilization protocol, GH11 endo-1,4-β-xylanase M1, from Trichoderma viride (Megazyme), was chosen as a model enzyme due to its robust structure and well-characterized hydrolytic activity. Its cleft-shaped active site provided accessibility to substrates while maintaining enzymatic functionality after covalent attachment to the functionalized CF surface.^30^ The functionalized CF_e_ as shown in Table 1 was incubated with GH11 xylanase, and enzyme retention was assessed across sequential wash steps (W1–5). “I” represents the initial enzyme activity, and “E” the residual activity in the solution after incubation with CF. Fraction “I” was measured once to represent free enzyme activity and showed product concentrations of 28 mM for xylose and 5.3 mM for cellobiose, while “E” and “W” samples were analyzed after each immobilization attempt and optimization. The immobilized enzyme (R) demonstrated hydrolytic activity, as evidenced by the production of xylobiose during the enzymatic assay on beechwood xylan (Megazyme) after 30 min of incubation at 40 °C^28^ (Figure 3). Enzymatic activity was determined by quantification of sugars released by using two complementary methods: high-performance anion-exchange chromatography with pulsed amperometric detection (HPAEC-PAD) and the 3,5-dinitrosalicylic acid (DNS) assay. HPAEC-PAD provided precise quantification of hydrolysis products (Figure 3A), while the DNS assay measured total reducing sugar concentrations (Figure 3B). Both methods confirmed the retention of enzymatic activity in the immobilized fraction, with xylose and xylobiose production being higher than in the final wash fractions (W5). The immobilization yield, efficiency, and activity recovery were calculated as described in the Materials and Methods section to evaluate the effectiveness of the protocol and the values are presented in Table 2. The yield, determined by quantifying the concentration of xylobiose released (which correlates with enzyme activity units), was 37.69%, indicating that a substantial portion of the enzyme was successfully immobilized on the CF surface. However, immobilization efficiency (21.62%) and activity recovery (8.15%) suggest partial enzyme inactivation or other factors contributing to these values. Sequential wash steps demonstrated the effective removal of unbound enzyme, while the immobilized fraction retained substantial activity. These findings indicate the covalent bonding between GH11 xylanase and the carboxylated CF surface. The stability of this model enzyme made it possible to optimize key parameters, such as buffer composition, enzyme loading, and washing steps, ensuring consistent results. These optimizations provide a basis for immobilizing more delicate enzymes, such as redox-active LPMOs.

**Figure 3:**
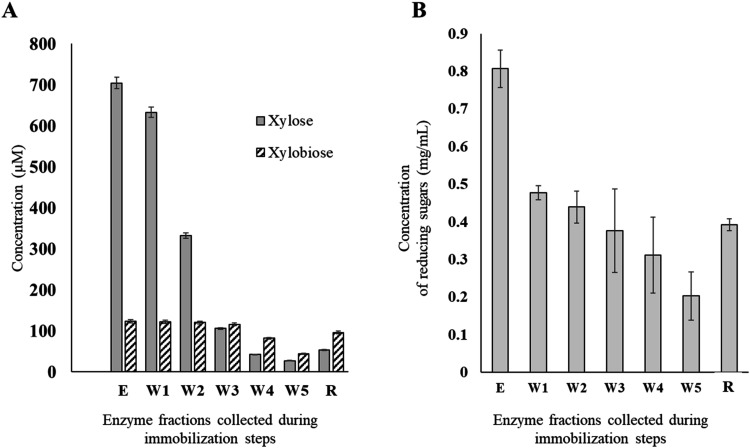
Quantification of enzymatic activity during the immobilization of GH11 xylanase on oxidized CF. (A) HPAEC-PAD analysis showing concentrations of xylose and xylobiose (μM) in enzyme fractions collected during immobilization (E, W1–W5) and from the immobilized CF fraction. (B) DNS assay results indicating the concentration of reducing sugars (mg/mL) in the same enzyme fractions. The trends confirm effective removal of unbound enzyme across wash steps (W1–W5) and retention of enzymatic activity in the immobilized CF (R) fraction.

**Table 2:** Optimization Parameters for Immobilization and Enzymatic Reaction Conditions

immobilized enzyme	COOH content on CF (mmol/g)	reaction time (h)	yield (%)	efficiency (%)	activity recovery (%)
Xylanase GH11	2.1	24	37.69	21.62	8.15
*Tth*LPMO9G	0.5	24	49.77	6.73	3.35
optimization of immobilization steps					
*Tth*LPMO9G	1.8	24	63.27	6.06	3.83
evaluation of stability of immobilized enzyme (second reaction cycle)					
*Tth*LPMO9G	1.8	4	51.55	15.01	7.74
*Tth*LPMO9G	1.8	24	75.16	1.71	1.28
*Tth*LPMO9G	1.8	72	76.40	0.76	0.58

### Immobilization of *Tth*LPMO9G on Oxidized CF and Biochemical Evaluation of Enzymatic Activity

The immobilization of *Tth*LPMO9G on oxidized CF was done following the same conditions as for xylanase immobilization; it was incubated with 0.2 mL of MES buffer (pH 6.0) containing 2 mg/mL *Tth*LPMO9G, fraction (I), at 4 °C for 24 h. The CF_d_ surface, as presented in Table 1, was chemically oxidized and incubated with 0.2 mL of MES buffer (pH 5.5) containing 2 mg/mL *Tth*LPMO9G, fraction (I), at 4 °C for 24 h. *Tth*LPMO9G has a pH optimum ranging from 5.5 to 7.5, as shown previously.^23^ Fraction I, representing the free enzyme reaction with PASC (0.2% w/v) as the substrate, was properly diluted and monitored once to confirm activity at a high enzyme concentration. The undiluted reaction would contain 13.4 μM *Tth*LPMO9G, corresponding to 1/5 of the initial 2 mg/mL enzyme solution (67.5 μM) added to the reaction. However, given the nonlinear relationship between enzyme concentration and product formation, as described in the first section of this study, the reaction was further diluted and recalculated based on the theoretical concentration of 13.4 μM. For the immobilized enzyme, 1/5 of the fractions (similar to the free enzyme) was used to estimate activity, despite the total immobilized or residual concentration in the fraction being unknown. Following immobilization, five sequential washing steps (W1–W5) were performed to remove unbound enzyme, and the immobilized fraction (R) was subjected to activity analysis. The study focused on two intermediate steps to evaluate the feasibility of the LPMO immobilization: (i) Biochemical analysis, where the immobilized enzyme was tested for its catalytic activity in the presence of an external reducing agent and PASC as a substrate under agitation, with CF serving only as an immobilization material and the focus be on assessing enzyme functionality after immobilization and, (ii) Electrochemical analysis, where the immobilized enzyme was tested in a bioelectrocatalytic setup with CF acting as the working electrode, aimed to determine its ability for direct electron transfer (DET) and enzymatic activity on PASC, as described in the following paragraph.

For the biochemical analysis, enzyme fractions were initially tested for peroxidase activity using the 2,6-dimethoxyphenol (2,6-DMP) assay as a quick detection method for LPMO activity (Table S2). It was observed that oxidized CF alone resulted in the appearance of a red absorbance signal, leading to false positives. The presence of copper further increased the background signal, making the assay unreliable.

So, the focus shifted to monitoring cellulose oxidation by the immobilized enzyme to assess its peroxygenase/monooxygenase activity, which was also the main goal of the study. The reactions were performed with PASC in MES buffer (50 mM, pH 5.5), using ascorbic acid (1 mM) as an electron donor, at 45 °C with 1000 rpm agitation for 24 h. Notably, the enzyme fractions from I, E, and W1–W5 were properly diluted for the reaction input, as the enzyme loading could not be quantified. This step was crucial to ensure consistency in the comparisons, particularly given that the kinetics would easily plateau at higher enzymatic concentrations, as shown in Figure 1. The products were quantified by HPAEC-PAD analysis. The activity assessment revealed that the free enzyme fractions (I) and (E) produced the highest levels of oxidized products, with a gradual decline observed across the wash fractions (W1–W5), revealing the removal of unbound enzyme. The immobilized CF fraction (R) demonstrated measurable oxidized product formation, though considerably lower than that of the free enzyme, indicating either that the enzyme was not effectively immobilized or that it was immobilized but lost a significant part of its stability (Figure S3). Further optimization efforts focused on varying the carboxyl group content on CF by increasing the oxidation time, as this parameter directly affected the immobilization efficiency and enzyme activity recovery, possibly because a higher carboxylate content could lead to more amide bonds and thus more covalent attachment with the enzyme. Samples with higher carboxyl densities (e.g., 1.8 mol −COOH/g CF) achieved improved immobilization yields (63.27%), as shown in Table 2 and Figure S3 compared to those with lower densities. However, activity recovery remained low (3.83%), likely due to enzyme inactivation during immobilization or reaction conditions. Furthermore, Table 2 shows that increasing immobilization yield often comes at the cost of reduced enzyme efficiency, and recovery, suggesting that strategies like increasing CF oxidation to enhance yield do not improve overall system performance. Importantly, attempts to reuse the immobilized enzyme in a second reaction set under the same conditions revealed a significant decline in performance. After initially assessing the immobilization yield and enzymatic activity, the immobilized enzyme was tested again to evaluate its stability over time or under reaction conditions. While the CF fraction retained some activity after the first reaction set, the efficiency and activity recovery dropped significantly in subsequent reaction sets (repetition tests), as evident when comparing the 24 h reactions from the first and second sets. In the second reaction set, reactions were tested at 4, 24, and 72 h to examine the impact of immobilization on enzyme kinetics. Notably, the 4 h reaction in the second set showed improved enzyme efficiency compared to the longer durations, despite the overall reduction in efficiency across the second cycle. Extending the reaction time during the second cycle (up to 72 h) did not restore enzyme efficiency or activity recovery, further emphasizing the inherent instability of the immobilized enzyme. These findings highlight the differences between soluble enzymes, which typically exhibit faster catalytic activity due to their unrestricted mobility, and immobilized enzymes.^28^

The observed loss of activity over repeated cycles suggests that structural instability of the immobilized enzyme and/or restricted substrate access within the CF matrix contributed to the reduced performance. Moreover, LPMOs are inherently susceptible to inactivation due to auto-oxidation and interactions with reactive oxygen species, which may further compromise their stability in immobilized systems. The immobilized *Tth*LPMO9G demonstrated initial activity but reduced reusability, highlighting the need for strategies to enhance stability and functionality. Future work could explore alternative immobilization methods and surface modifications to address these challenges and advance the application of immobilized LPMOs in bioelectrocatalysis.

### Electrochemical Evaluation of *Tth*LPMO9G Immobilized on Modified Carbon-Based Electrodes

For an immobilized enzyme to exhibit detectable electrochemical activity in cyclic voltammetry (CV), distinct redox peaks are expected. In the experiments, CV analysis (Figure 4) of immobilized *Tth*LPMO9G on chemically modified CF did not reveal clear enzymatic redox peaks. The signals closely resembled those of the blank control (CF without enzyme), indicating either the absence of enzymatic activity or weak activity masked by background currents. These results likely reflect the challenges associated with maintaining protein integrity and achieving optimal enzyme-electrode interactions during immobilization. To overcome the limitations of cyclic voltammetry (CV), Fourier-transformed alternating current voltammetry (FTacV) was employed, with interpretation of the results following the approach recently outlined by Lloyd-Laney et al.^31^ FTacV provided enhanced sensitivity and successfully detected harmonic patterns indicative of quasi-reversible electrochemical activity of the enzyme (Figure 5). The potential was cycled from 0.6 to −0.6 V with scan rate 5 mV/s, perturbation frequency 1 Hz and amplitude 400 mV. A quasi-reversible response has been recorded, as can be seen by the asymmetry and multiplicity of peaks at each harmonic. Based on the third harmonic, the principle cathodic peak is observed at *t* = 140 s, corresponding to −0.1 V, whereas the principle anodic peak is observed at *t* = 340 s, corresponding to 0.2 V. The blank reaction without enzyme in the FTacV analysis showed no distinct redox peaks, particularly from the third harmonic onward, indicating the absence of electrochemical activity in the system without *Tth*LPMO9G (Figure S4). Following the evaluation of the electrochemical activity of *Tth*LPMO9G immobilized on modified CF to detect redox cycles, the study proceeded to its final stage. Figure 6 presents a schematic representation of the immobilization process for *Tth*LPMO9G on functionalized CF and its potential application in an electrochemical system for cellulose substrate oxidation. However, direct electron transfer coupled with enzymatic activity was attempted but proved challenging under the tested conditions. The challenges of the system might include: (i) Protein integrity and stability. Biochemical characterization revealed a significant loss of protein integrity and stability for the immobilized enzyme. Covalent immobilization, while providing strong binding to the CF surface, likely disrupted the enzyme’s native structure and reduced its activity. This finding aligns with observations for other oxidoreductases, where covalent attachment resulted in structural alterations and substantial activity loss.^32^ (ii) Limited electrochemical activity due to active site proximity to the electrode. From an electrochemical perspective, the efficiency of DET depends on the proximity of the enzyme’s active site to the electrode surface, ideally within a few angstroms. In the current design, *Tth*LPMO9G contains seven lysine residues that could form covalent bonds with the CF. This creates multiple possible orientations, making it difficult to position the active site close enough to the electrode for efficient electron transfer. The undefined orientation further complicates the ability to achieve direct electron provision. Previous studies have highlighted that the distance dependence of the electron transfer rate plays a key role in determining why certain experimental conditions enable direct electron transfer.^33^ They reported that electron transfer rates between the enzyme and the electrode surface decrease exponentially as the distance increases. (iii) Combined biochemical and electrochemical issues. The simultaneous interplay of the above problems exacerbates the overall system’s inefficiency. The instability of the immobilized enzyme, along with the difficulty in facilitating electron transfer, poses combined challenges that limit the system’s effectiveness. (iv) The reaction system’s design presented significant challenges due to its larger electrochemical cell volume, with 30 mL of 0.1% (w/v) cellulose as the substrate—substantially exceeding the typical lab-scale LPMO reaction volumes. LPMO-generated oxidized oligosaccharides are typically detected in the micromolar range. However, HPAEC-PAD analysis of the soluble reaction products revealed a flat chromatogram (data not shown), indicating that oxidized cello-oligosaccharides were below the detection limit under these experimental conditions. (v) Substrate attachment to the modified CF. Another issue observed during the experiments was the unexpected affinity of the insoluble cellulose substrate (PASC) to the modified CF. Even under agitation, visual observation suggested that the cellulose fibers adhered to the CF. This adhesion could stem either from the ability of the carbohydrate-binding module (CBM) of *Tth*LPMO9G to bind onto cellulose, which would indicate a functional interaction or physicochemical phenomena, since the modified CF’s surface properties might cause cellulose to form a layer or coat on the CF, trapping the enzyme, substrate, or products. In case the adhesion is CBM-mediated, this could be a positive outcome for creating a localized reaction zone. However, if it results from physicochemical interactions, it could further reduce protein integrity, exacerbate substrate trapping, or hinder product release, making product identification and quantification even more challenging. The challenges of enzyme instability, the distance between the active site and the electrode, product dilution in large reaction volumes, and substrate adhesion to the modified CF collectively prevented the envisioned DET system from functioning as intended. To address these issues, future work should focus on improving immobilization methods to preserve enzyme activity and stability, designing electrodes that position the active site closer to the surface to enhance electron transfer and facilitate product detection, and addressing substrate adhesion to ensure effective substrate conversion and product release. Another crucial point to consider is the conformation of the active site. It is well-documented that the substrate, cellulose, plays a role in shielding the active site, enabling the histidine brace to adopt the configuration required for catalyzing monooxygenase/peroxygenase reactions.^34^ In an immobilized environment, it can be hypothesized that the active site may remain more stably shielded, potentially increasing enzyme stability and reducing the risk of inactivation. However, in a scenario where the active site is oriented toward a conductive electrode to facilitate efficient electron transfer, the accessibility of insoluble cellulose to the active site might be restricted. This limitation could shift the enzymatic activity toward oxidase reactions rather than monooxygenase/peroxygenase reactions. Another open question is whether the LPMO to be immobilized should include a CBM or not. While the presence of a CBM could potentially stabilize the enzyme,^34,35^ it may also increase the rigidity of the system. In an immobilized setup, where the LPMO needs to oxidize an insoluble polysaccharide, the limited mobility caused by the CBM might further hinder substrate accessibility and decrease oxidation efficiency. These are open questions that need to be addressed. The answers will ultimately determine whether the innovative and biotechnologically useful design of DET to LPMO systems for cellulose oxidation is practically achievable or not. Despite these hurdles, the study offers valuable lessons for developing improved bioelectrochemical systems using LPMOs for sustainable biomass conversion. These intermediate steps provide insights, and once are optimized, the final goal of providing electrons directly to the LPMO active site via the electrode in the presence of cellulose can be achieved.

**Figure 4:**
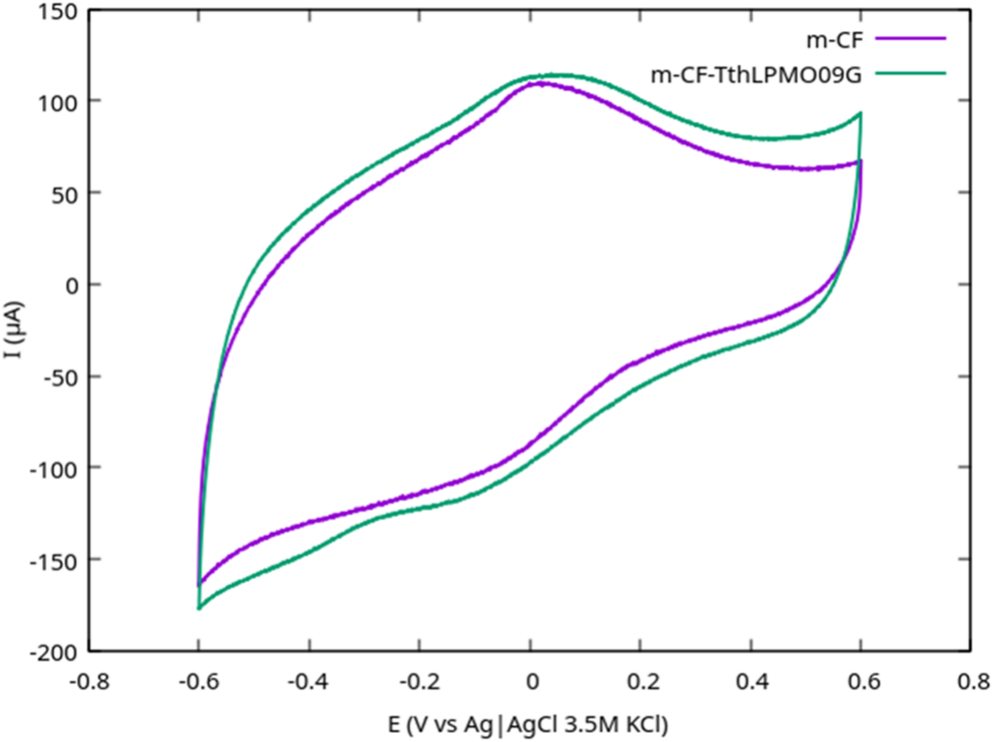
Cyclic voltammograms recorded within a potential range of [−0.8 to 0.8 V] at a scan rate of 10 mV/s for CF substrates in sodium acetate buffer (pH 5.5). The CF substrates were chemically oxidized and functionalized with EDC/NHS for enzyme immobilization. The control carbon felt (CF) without enzyme and CF with immobilized *Tth*LPMO9G did not show distinguishable redox peaks, suggesting weak or masked electrochemical activity of the immobilized enzyme under these experimental conditions.

**Figure 5:**
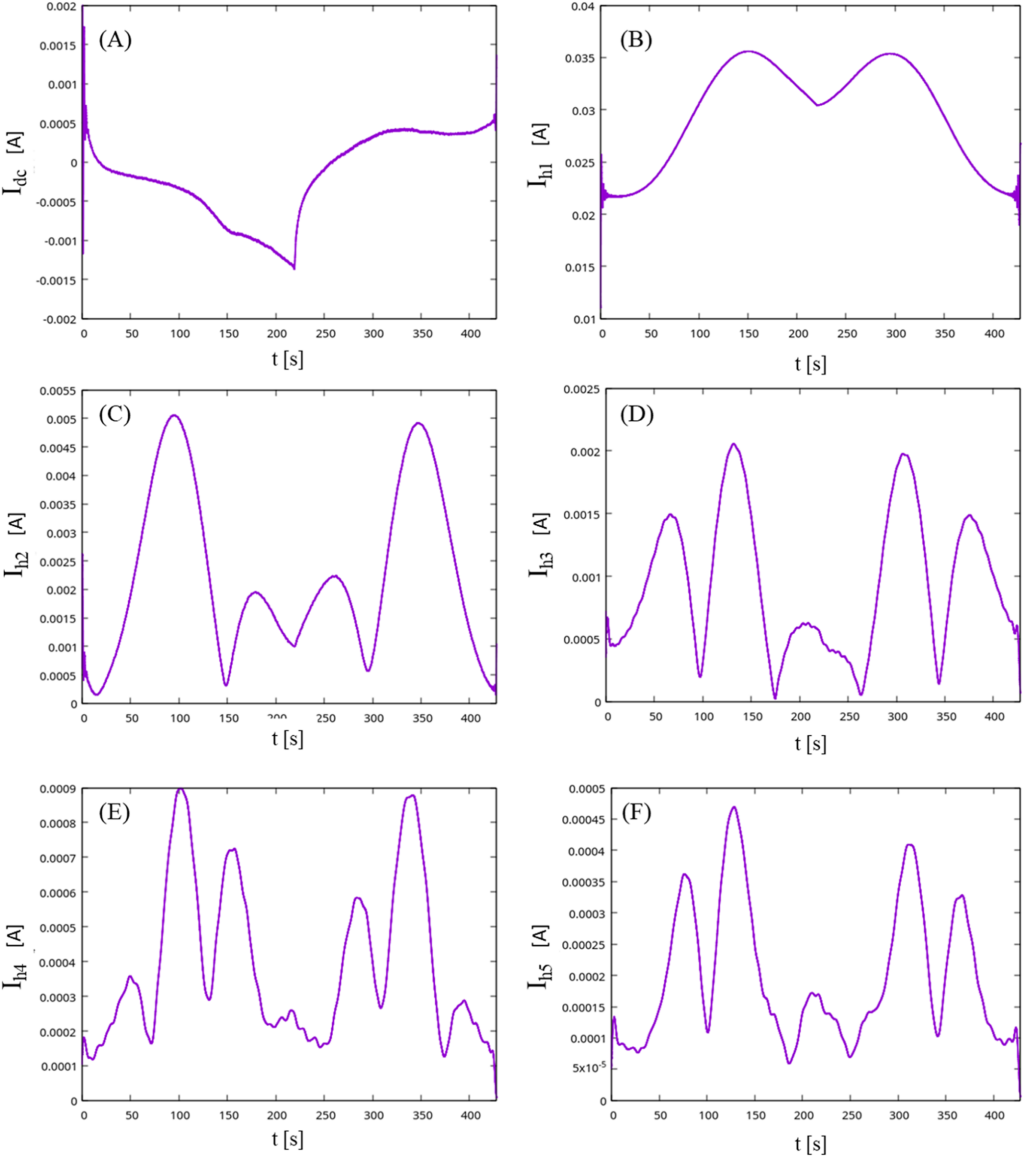
FTacV diagrams of the direct current, 1st, 2nd, 3rd, 4th and 5th harmonics for the modified CF electrode with immobilized *Tth*LPMO9G, recorded under conditions of 400 mV amplitude, 1 Hz frequency, and 5 mV/s scan rate. While cyclic voltammetry did not reveal electrochemical activity of the enzyme on CF, FTacV analysis indicated quasi-reversible redox activity. Panels (A–F) correspond to the direct current (DC) component and the first through 5th harmonics, respectively.

**Figure 6:**
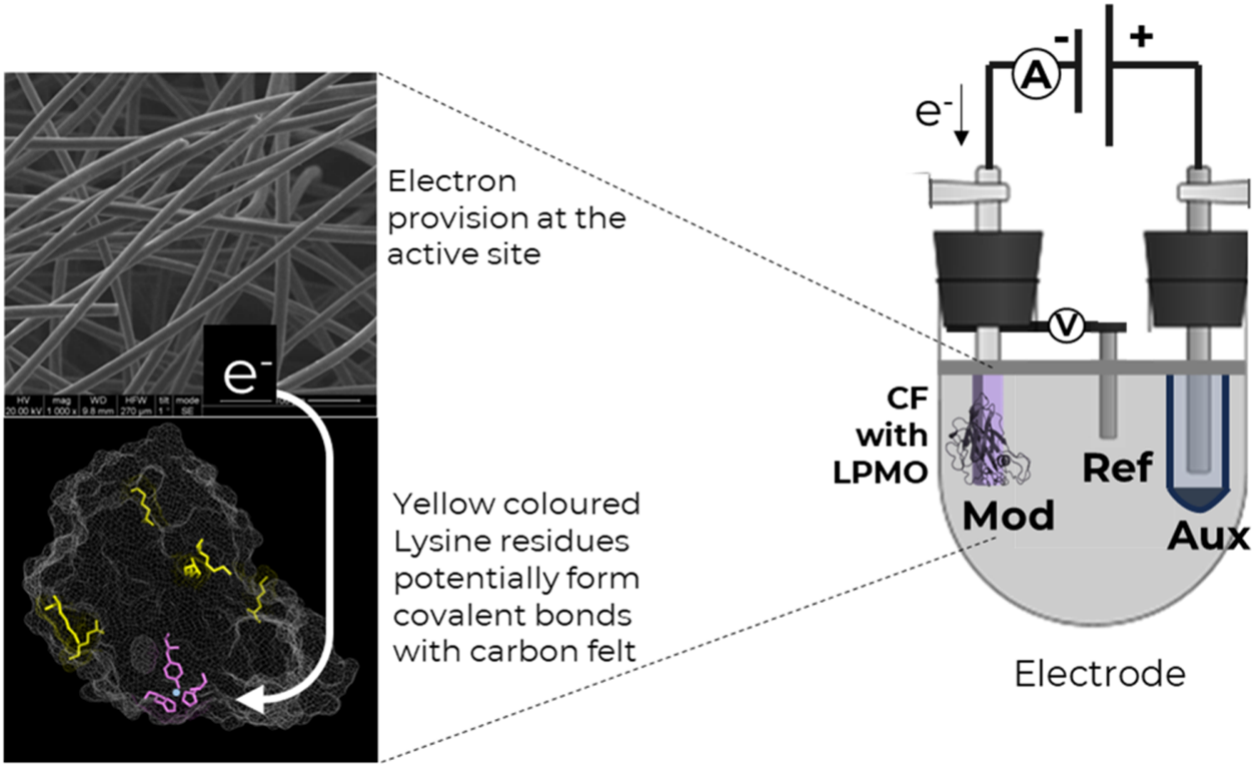
Schematic representation of the immobilization of *Tth*LPMO9G on functionalized CF and its potential application in an electrochemical system. The left panel shows the structure of oxidized CF, highlighting its high surface area and conductive properties. The LPMO structure is depicted with the purple active site containing copper and yellow lysine residues, which are assumed to form covalent bonds with the functionalized CF through reactive carboxyl groups. The right panel illustrates the theoretical design of a bioelectrochemical setup, where the immobilized enzyme on CF serves as the working electrode (Mod) in a three-electrode system, enabling controlled electron transfer to the LPMO active site for catalytic activity.

## Materials and Methods

### Preparation of Carbon-Based Electrodes

Carbon-based electrodes were prepared using carbon fiber bundles approximately 10 cm in length. The carbon fibers were threaded through a glass Pasteur pipet, to act as a current collector. The glass tube provided insulation, while the carbon fibers were secured in place using parafilm. The final 1 cm of the carbon fiber was left outside the glass tube to serve as the active electrode area. This exposed section was thoroughly washed with acetone and deionized water to remove contaminants. It was then uniformly coated with DUE-CI ELECTRONIC Graphite Spray N-77 and allowed to air-dry. After drying, the fibers were washed again with acetone followed by deionized water.

The graphite-coated section was immersed in a Nafion solution for entrapment, which included either the enzyme (final concentration of 2 mg/mL) ferrocene or potassium ferricyanide as controls. The fibers were incubated in the solutions for 2 h to ensure proper entrapment. After incubation, the fibers were removed, air-dried, and stored for use in subsequent experiments. The remaining length of the carbon fiber within the glass tube served as the conductive path for current collection, ensuring an insulated and functional electrode design for electrochemical applications.

Carbon felt electrodes were also prepared as active electrode materials (AvCarb G200, PAN-based). These electrodes were attached to carbon fibers that served as current collectors. Carbon fiber bundles, approximately 10 cm in length, were used as current collectors, as shown in Figure 7. The carbon felt was threaded through a glass Pasteur pipet, to provide insulation, while the carbon fibers were secured in place using parafilm. The surface morphology was estimated via scanning electron microscopy (SEM) using a Philips Quanta Inspect (FEI Company) microscope with W (tungsten) filament 25 kV equipped with equipped with an Energy-Dispersive X-ray Spectrophotometer (EDS) EDAX Genesis (AMETEX Process and Analytical Instruments). The carbon felt, underwent chemical modification to introduce carboxylic (−COOH) groups on its surface, enabling covalent bonding with enzymes. Carbon felt pieces were immersed in a mixture of concentrated sulfuric acid (95%) and nitric acid (65%) in a 3:1 volume ratio. This mixture was heated at 80 or 120 °C for durations ranging from 8 to 64 h under constant agitation. After oxidation, the carbon felt was extensively rinsed with deionized water until the pH and conductivity of the final rinses matched those of deionized water. The oxidized carbon felt was then dried at 60 °C for 2.5 h. Following chemical oxidation, the carbon felt was divided into two halves. One half of the carbon felt was attached to the glass Pasteur pipet tube to serve as the active electrode. As in the previous system, carbon-based electrodes were prepared using carbon fiber bundles approximately 10 cm in length. The carbon fibers were threaded through a glass Pasteur pipet to act as a current collector. In this setup, the final 1 cm of the electrode was carbon felt, which was attached to the carbon fibers. This design ensured compatibility between the carbon felt and carbon fibers, minimizing interference in the electrochemical measurements. While the carbon fibers continued to act as the current collector, the carbon felt was considered the active electrode material in this system. This piece weighed approximately 20 mg, with dimensions of 7.5 mm × 5 mm × 2.5 mm. The other half was used to quantify the introduced carboxylic groups via conductometric titration. The dispersion was continuously titrated with 0.01 M NaOH, and the carboxylate content (*C*_COOH_, mmol/g) was calculated using the equation: *C*_COOH_ = (*V*_2_ – *V*_1_) × *C*_NaOH_/DW_sample_, where *V*_2_ – *V*_1_ represents the volume of NaOH at the equivalence conductivity point, *C*_NaOH_ is the NaOH concentration (0.01 M), and DW_sample_ is the sample’s dry weight (g). After oxidation, the CF was rinsed with deionized water until the pH and conductivity of the final rinses matched those of deionized water. The oxidized CF was subsequently dried at 60 °C for 2.5 h. The oxidized CF was then treated with 1-ethyl-3-(3-(dimethylamino)­propyl)­carbodiimide (EDC) and *N*-hydroxysuccinimide (NHS) in MES buffer (0.2 M, pH 5.0) to activate the carboxylic groups for enzyme immobilization.^7^ The activation reaction was carried out at room temperature for 16 h with gentle stirring. The oxidized carbon felt was treated with 1-ethyl-3-(3-(dimethylamino)­propyl)­carbodiimide (EDC) and *N*-hydroxysuccinimide (NHS) in MES buffer (0.2 M, pH 5.5) to activate the carboxylic groups for enzyme immobilization. This activation reaction was carried out at room temperature for 16 h with stirring. This process ensured that the carbon felt electrodes were properly functionalized and prepared for enzymatic immobilization and subsequent electrochemical applications.

**Figure 7:**
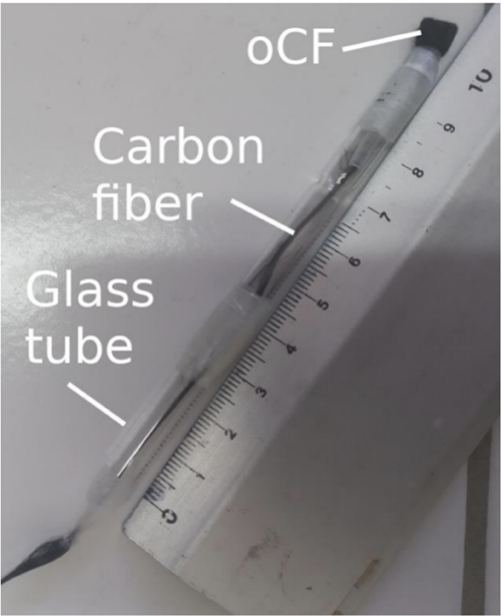
Assembly of the electrochemical electrode setup. Oxidized carbon felt (oCF) is connected to a carbon fiber bundle threaded through a glass Pasteur pipet for insulation. The carbon fiber serves as the current collector.

### Enzyme Immobilization and Activity Assessment

*Tth*LPMO9G was expressed and characterized as previously described.^22^ The en-do-1,4-β-xylanase M1 from T. viride (GH11) was purchased from Megazyme and used to standardize the immobilization protocol. Activated CF pieces were incubated overnight with a concentrated enzyme solution (2 mg/mL) in MES buffer (0.2 M, pH 5.5) at 4 °C for 16–24 h without stirring. The low-temperature incubation was chosen to maintain system stability, prevent enzyme destabilization, and allow sufficient time for covalent bond formation where applicable. Following immobilization, the CF was washed with MES buffer or sodium acetate buffer (0.1 M, pH 5.5) at 45 °C under agitation (800 rpm) to remove unbound enzyme. The washing steps were performed at 45 °C to closely match the subsequent reaction conditions, ensuring that any loosely adsorbed or nonspecifically bound enzyme would be released during the washes rather than during the enzymatic assay. Performing the washes at a lower temperature could have led to the retention of weakly bound enzyme, which might subsequently detach when exposed to reaction conditions, potentially confounding activity measurements. This approach ensured that only the covalently immobilized enzyme remained on the CF before enzymatic and electrochemical analysis. The effectiveness of immobilization was assessed using three key parameters: immobilization yield, immobilization efficiency, and activity recovery.^28^ These parameters were calculated by comparing the activity of the immobilized enzyme on cellulose substrates with the activity remaining in solution and wash fractions. Immobilization yield represents the percentage of total enzyme activity from the starting solution that is immobilized on the support and was calculated as

1 immobilizationyield(%)=[(I−(E+W1+W2+W3+W4+W5))/I]×100

where I is the initial enzyme activity, E is the residual enzyme activity in the solution after incubation with CF, and W1 to W5 are the activities in the wash fractions. The immobilized activity is determined by subtracting the total residual activity from the starting activity. Immobilization efficiency describes the percentage of bound enzyme activity that remains active and was calculated as

2 immobilizationefficiency(%)=[R/(I−(E+W1+W2+W3+W4+W5))]×100

where R is the observed activity of the immobilized enzyme on carbon felt. This metric highlights the effectiveness of the immobilization process in retaining enzymatic activity on the support. Activity recovery quantifies the overall success of the immobilization process by comparing the observed activity of the immobilized enzyme to the starting enzyme activity

3 activityrecovery(%)=(R/I)×100

To ensure accurate measurements, the residual enzyme activity (E) and the activities in the wash fractions (W1 to W5) were measured after the immobilization process. In addition, a blank experiment was conducted to account for potential free enzyme deactivation under immobilization conditions. Both enzyme activity and protein concentration in the supernatant were monitored to verify that the enzyme remained active during the process and to ensure accurate calculation of enzyme loading on CF. Protein-based measurements alone were avoided to prevent misinterpretation, especially when crude protein mixtures were used. This approach provides a consistent framework for evaluating the success of enzyme immobilization while addressing potential issues such as enzyme deactivation and incomplete binding.

The activity of immobilized *Tth*LPMO9G was tested on PASC using sodium acetate buffer (50 mM, pH 5.5), with 1 mM ascorbic acid as the electron donor. Reactions were conducted at 45 °C with stirring (1000 rpm) for 24 h. Reaction products were analyzed by HPAEC-PAD, Dionex, with the quantification of the C1-oxidized cello-oligosaccharides released employed for determination of LPMO activity. The system was equipped with a CarboPac PA1 guard column (2 mm × 50 mm) and an analytical CarboPac PA1 column (2 mm × 250 mm). Two mobile phases were used: A (0.1 M NaOH) and B (1 M sodium acetate in 0.1 M NaOH). For the analysis of C1-oxidized cello-oligosaccharides, a 40 min elution method was applied at a flow rate of 1 mL/min, as described previously.^36^

The standardized immobilization protocol was first optimized using GH11 xylanase from T. viride (Megazyme). All fractions and the immobilized enzyme were incubated in reaction mixtures containing 0.5 mg/mL beechwood xylan (Megazyme) in 50 mM sodium acetate buffer (pH 5.0) at 50 °C for 30 min. Reducing sugars were measured using the DNS assay, as previously described.^37^ Xylose and xylobiose were also quantified using HPAEC-PAD (Dionex ICS5000) with a CarboPac PA1 guard column (2 mm × 50 mm) and analytical column (2 mm × 250 mm). Eluent A was 0.1 M NaOH and eluent B was 1 M sodium acetate in 0.1 M NaOH. Xylose and xylobiose were detected at 5 and 7 min, respectively.

### Electrochemical Measurements

Voltammetric experiments were conducted in a single-compartment, three-electrode cell. A titanium wire with a diameter of 1 mm served as the counter electrode, and an Ag|AgCl (KCl saturated, +0.197 V vs NHE) was used as the reference electrode. The cell contained an aqueous solution of approximately 60 mL, consisting of either 1 M Na_2_SO_4_ (98%, Merck) as the supporting electrolyte or 50 mM MES buffer (pH 5) (PENTA). Cyclic voltammetry (CV) measurements were performed using a PalmSens4 Potentiostat. All solutions were deaerated for at least 20 min prior to the experiments to prevent oxygen reduction on the electrode surface or potential catalytic currents, particularly if the LPMO catalytic mechanism involves oxygen reacting with the enzyme. Nitrogen gas was continuously purged over the solution during measurements to maintain anaerobic conditions at room temperature.^8^

Fourier-transformed alternating current voltammetry (FTacV) experiments were conducted under similar conditions to those used for CV but employed a PAR 263A potentiostat in conjunction with an AFG 5101 Tektronix programmable arbitrary function generator. Data from FTacV experiments were analyzed using a custom in-house program, which enabled precise identification and quantification of harmonic components to investigate the electrochemical behavior of the LPMO-modified electrodes.^8^

## Conclusions

This study investigated the entrapment and immobilization of *Tth*LPMO9G on oxidized CF support, contributing to the largely unexplored field of LPMO immobilization for bioelectrochemical applications. While DET and efficient catalytic performance remain challenging due to steric hindrance, enzyme orientation and substrate adhesion, this work provides a valuable experimental framework for future studies. Enhanced CV performance of *Tth*LPMO9G entrapped in Nafion polyelectrolyte, as well as FTacV detected electrochemical activity of the *Tth*LPMO9G immobilized onto modified CF suggest potential for integrating LPMOs into electrochemical platforms. Biochemical characterization confirmed successful enzyme attachment but revealed limitations in activity recovery and protein stability, highlighting the complexities of immobilizing such fragile enzymes. Despite these limitations, the study establishes a foundation for further development of immobilization strategies tailored to LPMOs.

## Supplementary Material



## Data Availability

All data supporting the conclusions of this article are included in the manuscript and its additional files. Samples of materials produced in the current work are available from the corresponding author upon reasonable request.

## Notes

Control HPAEC-PAD chromatogram of PASC incubated with 4 mM ascorbic acid in the absence of *Tth*LPMO9G (Figure S1); SEM images of CF (Figure S2); the quantification of oxidized cello-oligosaccharides produced during LPMO immobilization and subsequent activity testing under different CF functionalization levels and over a time course (Figure S3); results from the FTacV analysis of the blank CF electrode (Figure S4); the physical properties of the carbon felt (CF, AvCarb G200) used for enzyme immobilization are summarized (Table S1), and results from the 2,6-DMP assay, including immobilized LPMO samples, carbon felt alone, and copper controls are presented (Table S2) (PDF)
