# Supplementary material for: Immobilization of TthLPMO9G on Carbon Felt for Potential Electrochemical Applications
Source: ACS Omega. 2025 May 12;10(20):20895–906. doi: 10.1021/acsomega.5c02275 (PMC12120643; doi:10.1021/acsomega.5c02275)
Supplement: Supplementary file 1 [file ao5c02275_si_001.pdf]

Immobilization of *Tth*LPMO9G on Carbon Felt for potential Electrochemical Applications

Koar Chorozian[1], Anthi Karnaouri[2], Theodora Kouvarati[3], Antonis Karantonis[3], Evangelos Topakas[1*]*

[1] IndBioCat Group, Biotechnology Laboratory, School of Chemical Engineering, National Technical University of Athens, Athens, Greece

[2] Laboratory of General and Agricultural Microbiology, Department of Crop Science, Agricultural University of Athens, Athens, Greece

[3] Laboratory of Physical Chemistry, School of Chemical Engineering, National Technical University of Athens, Athens, Greece

**Corresponding author: vtopakas@chemeng.ntua.gr*

**Corresponding author: antkar@mail.ntua.gr*

Supporting Information

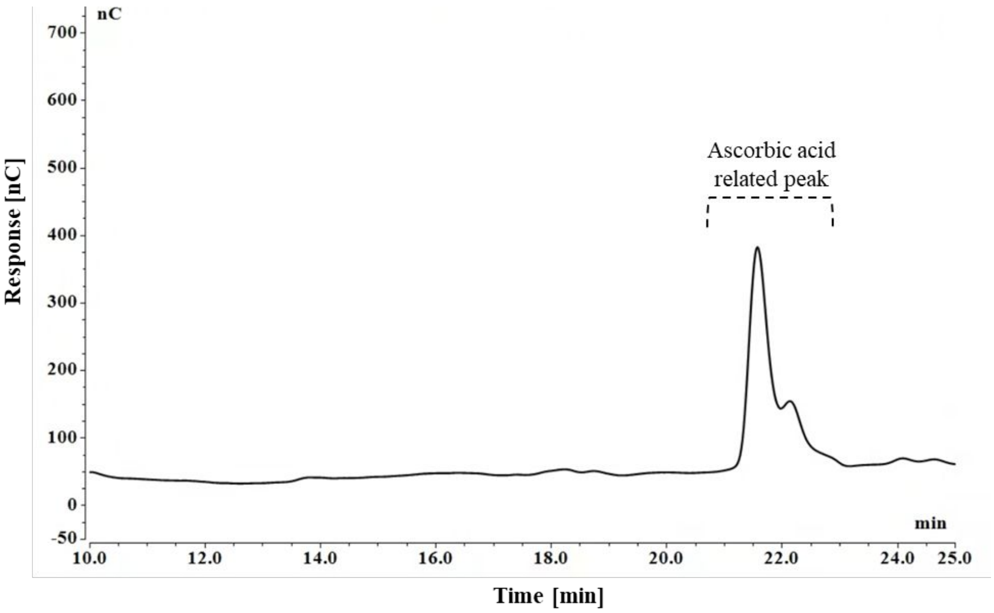

Figure S1. Control reaction with 4 mM ascorbic acid in the absence of *Tth*LPMO9G. HPAE-PAD analysis of PASC incubated with 4 mM ascorbic acid, without enzyme. No C1-oxidized products were detected. The ascorbic acid related peak appears at ~22 min.

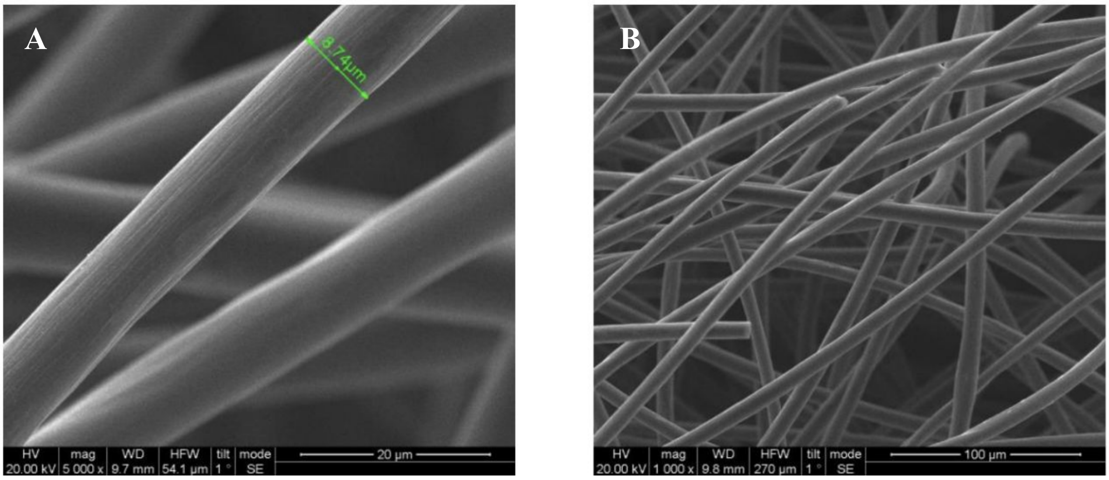

Figure S2. Scanning electron microscopy images of CF (AvCarb G200). (**A**) Close-up of individual fibers, showing an average diameter of 8.74 µm. (**B**) A broader view of the dense fibrous network.

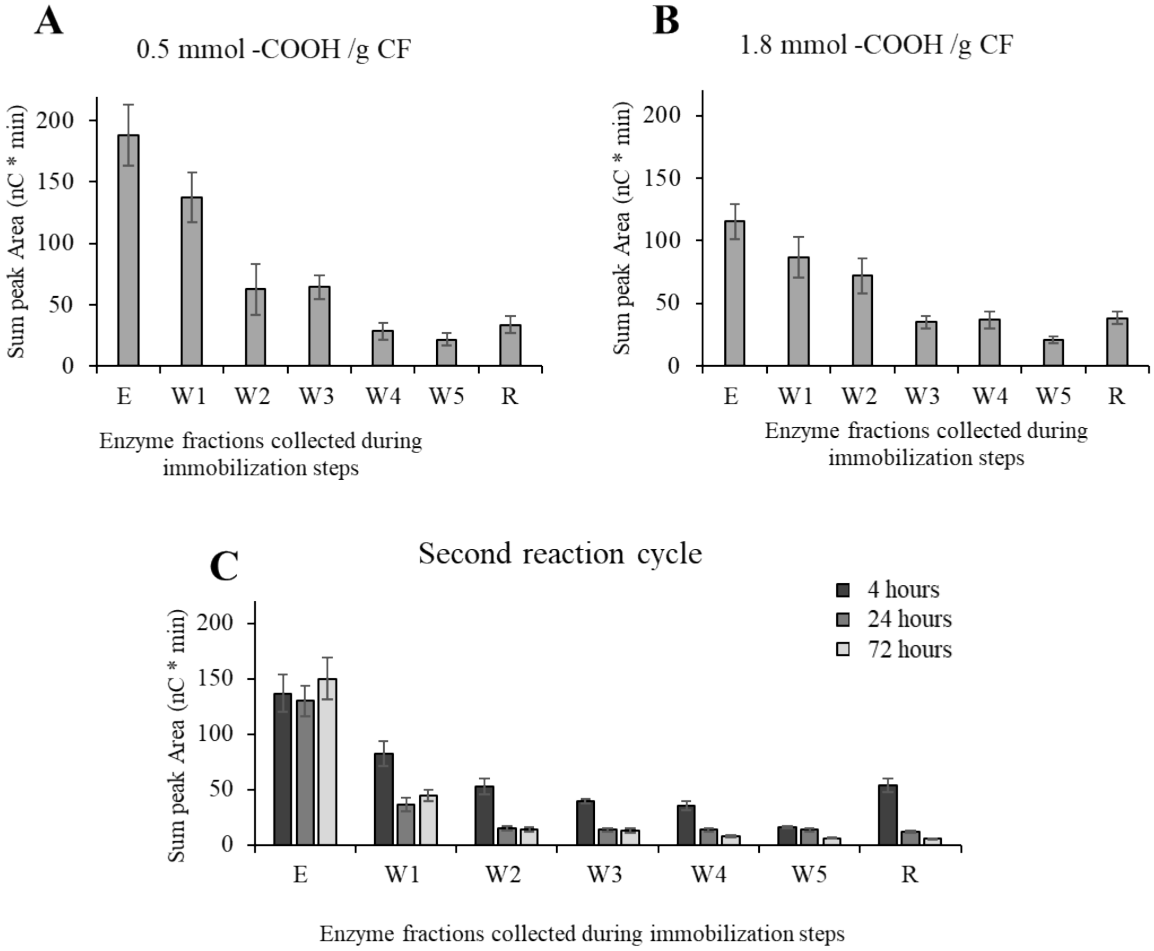

Figure S3. Quantification of oxidized cello – oligosaccharides during the immobilization of *Tth*LPMO9G on carbon felt and its activity evaluation. Reactions were performed with PASC, 0.2% w/v and ascorbic acid 1 mM as the reducing agent. (**A**) Activity assessment during immobilization on CF functionalized with 0.5 mmol -COOH/g CF. The sum peak area of C1-oxidized products was quantified by HPAEC-PAD for enzyme fractions collected at each step (E: enzyme remained in solution, W1–W5: sequential wash fractions, R: immobilized enzyme). (**B**) Activity assessment during immobilization on CF functionalized with 1.8 mmol -COOH/g. (**C**) Stability evaluation of the immobilized enzyme on 1.8 mmol -COOH/g CF during a second reaction cycle. The immobilized enzyme (R) was tested with PASC over a time course of 4, 24, and 72 h.

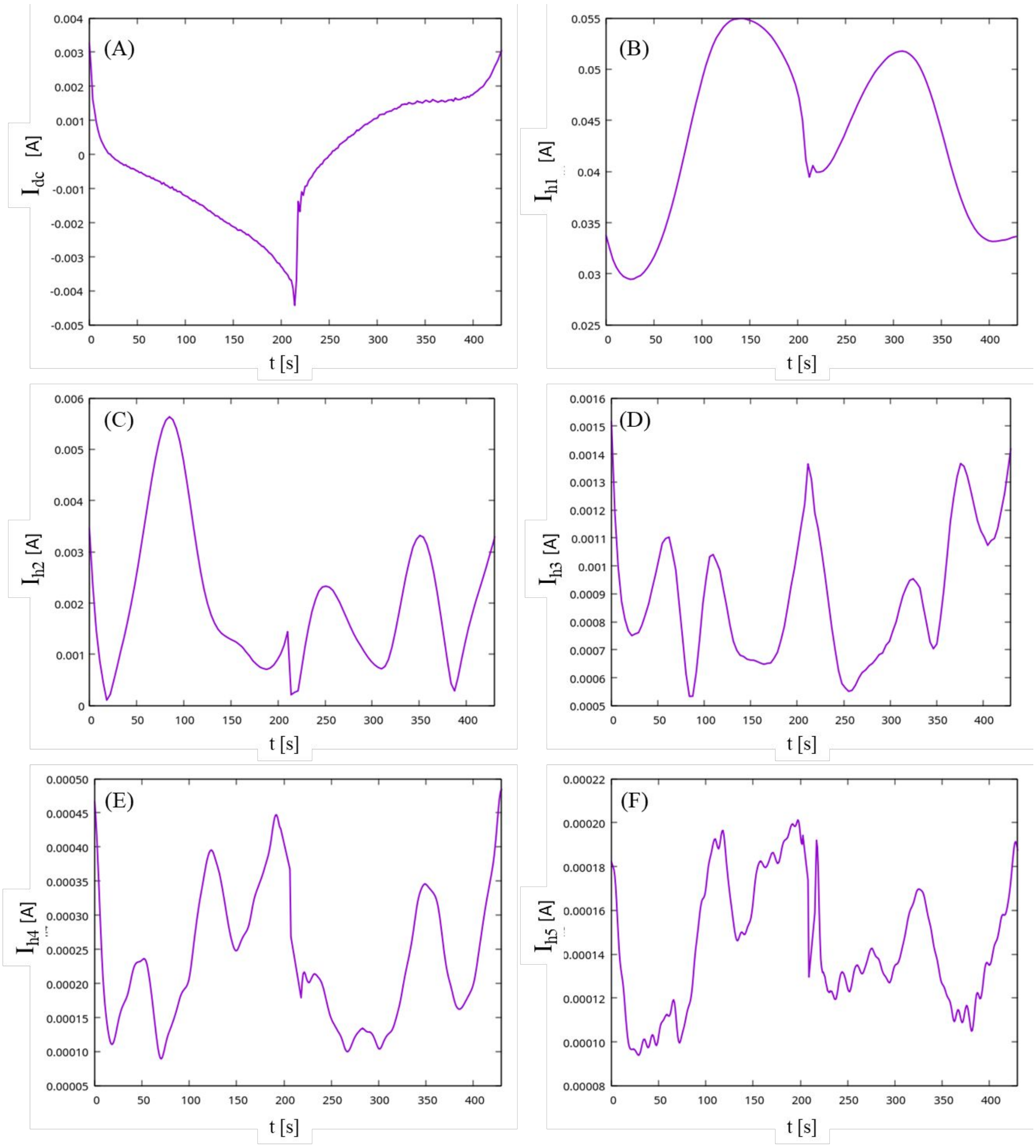

Figure S4. FTacV diagrams of the direct current and 1st to 5th harmonics for the modified CF electrode without immobilized enzyme (blank control), recorded at 400 mV amplitude, 1 Hz frequency, and 5 mV/s scan rate. From the 3rd harmonic onward, characteristic patterns indicative of redox activity are not observed.

Table S1. Properties of carbon felt (CF).

**Property**	**Value**	**Unit**
Thickness	6.5	mm
Density	0.07	g/cm^3
Weight per area	480	g/m^2
Electrical resistance	< 3	Ω·mm
Carbon content	99 (minimum)	%
Ash content	< 0.2	%

Table S2. 2,6-DMP assay results for LPMO immobilized on CF, CF alone and copper (Cu) control reactions.

**Sample**	**Blank**	**with LPMO**	**with Cu**	**with Cu and LPMO**
W5	2.46 ± 0.7 U/g	4.73 ± 1.1 U/g	4.00 ± 0.8 U/g	9.60 ± 1.3 U/g
R	3.8 ± 1.1 U/g	9.60 ± 1.4 U/g	3.93 ± 0.9 U/g	4.53 ± 1.2 U/g
